# Identification of protein stability determinants in chloroplasts

**DOI:** 10.1111/j.1365-313X.2010.04268.x

**Published:** 2010-07-09

**Authors:** Wiebke Apel, Waltraud X Schulze, Ralph Bock

**Affiliations:** Max-Planck-Institut für Molekulare PflanzenphysiologieAm Mühlenberg 1, D-14476 Potsdam-Golm, Germany

**Keywords:** protein stability, protein degradation, N-end rule, chloroplast, plastid transformation, *Escherichia coli*

## Abstract

Although chloroplast protein stability has long been recognised as a major level of post-translational regulation in photosynthesis and gene expression, the factors determining protein stability in plastids are largely unknown. Here, we have identified stability determinants *in vivo* by producing plants with transgenic chloroplasts that express a reporter protein whose N- and C-termini were systematically modified. We found that major stability determinants are located in the N-terminus. Moreover, testing of all 20 amino acids in the position after the initiator methionine revealed strong differences in protein stability and indicated an important role of the penultimate N-terminal amino acid residue in determining the protein half life. We propose that the stability of plastid proteins is largely determined by three factors: (i) the action of methionine aminopeptidase (the enzyme that removes the initiator methionine and exposes the penultimate N-terminal amino acid residue), (ii) an N-end rule-like protein degradation pathway, and (iii) additional sequence determinants in the N-terminal region.

## Introduction

Plastids arose through endosymbiosis of a formerly free-living cyanbacterium, and have therefore retained numerous prokaryotic features in their gene expression machinery, for example organization of genes into operons and translation on 70S-type ribosomes. However, over the course of evolution, plastids have also acquired numerous novel features that are clearly not of eubacterial origin and make the regulation of plastid gene expression quite complex. These include the presence of multiple RNA polymerases and promoter types (Liere and Börner, 2007), the prevalence of post-transcriptional control of gene expression (Schmitz-Linneweber and Small, 2008), the processing of polycistronic into monocistronic mRNAs (Zhou *et al.*, 2007), and the utilisation of RNA editing as an additional RNA maturation mechanism (Bock, 2000; Schmitz-Linneweber and Barkan, 2007). Plastid gene expression is also extensively controlled at the post-translational level, mainly via regulated protein complex assembly and proteolysis (Adam, 2000, 2007; Kanervo *et al.*, 2007). For example, protein degradation plays a crucial role in the replacement of photo-oxidatively damaged photosynthesis proteins (Sakamoto *et al.*, 2003; Zaltsman *et al.*, 2005; Kapri-Pardes *et al.*, 2007; Park *et al.*, 2007), as well as in removal of superfluous subunits of the multiprotein complexes involved in photosynthetic electron transport (Choquet and Vallon, 2000; Majeran *et al.*, 2000; Drapier *et al.*, 2007).

Biochemical and genetic studies have unravelled several plastid proteolytic activities (reviewed by Adam, 1996, 2000; Sakamoto, 2006; Adam *et al.*, 2006). The plastid-localized proteases identified so far are homologous to eubacterial proteases, and include the ATP-dependent proteases Clp, FtsH and Lon and the ATP-independent Deg protease (Shanklin *et al.*, 1995; Lindahl *et al.*, 1996; Itzhaki *et al.*, 1998; Haußkühl *et al.*, 2001). Interestingly, many of these proteases (and/or their subunits) are encoded by multi-gene families in the nuclear genome, and the emerging differential functions of the individual family members suggest an intricate regulatory network of protein degradation in plastids (Peltier *et al.*, 2004; Zaltsman *et al.*, 2005; Rudella *et al.*, 2006; Kapri-Pardes *et al.*, 2007; Kim *et al.*, 2009).

Although our knowledge about plastid proteases has progressed in the last decade at a rapid pace, almost nothing is known about stability or instability determinants within the substrate proteins. As in bacteria, plastid-encoded proteins are post-translationally processed by N-terminal deformylation and excision of the initiator methionine (Giglione *et al.*, 2000, 2003; Giglione and Meinnel, 2001). Both deformylation (by the enzyme peptide deformylase, PDF) and N-terminal Met excision (by the enzyme Met aminopeptidase, MAP) appear to be required for proper chloroplast development (Giglione *et al.*, 2003, 2004; Moon *et al.*, 2008), and removal of the initiator Met has been suggested to influence the stability of plastid proteins (Giglione *et al.*, 2003). Nuclear-encoded proteins that are targeted to plastids are also post-translationally processed by post-import cleavage of the transit peptide harbouring the targeting information. It seems reasonable to assume that, as in bacteria and eukaryotes, sequence motifs and/or structural features of a given plastid protein influence its half life.

The best-known factor determining the turnover time of proteins in all organisms investigated to date is described by the so-called N-end rule. This rule correlates the half life of a protein with the identity of its N-terminal amino acid (Varshavsky, 1996; Mogk *et al.*, 2007). Similar but distinct versions of the N-end rule operate in prokaryotes and eukaroytes. In eukaryotes, the N-end rule pathway of protein degradation is part of the ubiquitin system, in that proteins carrying a destabilising residue at their N-terminus are ubiquitinated and degraded by the 26S proteasome (Varshavsky, 1996; Tasaki and Kwon, 2007). In contrast, the bacterial version of the N-end rule pathway utilises the Clp protease (Tobias *et al.*, 1991; Erbse *et al.*, 2006), and the ClpAP adaptor protein ClpS appears to play a crucial role in substrate recognition (Román-Hernández *et al.*, 2009; Schmidt *et al.*, 2009).

The eukaryotic and prokaryotic versions of the N-end rule also differ in the hierarchical order of stabilising and destabilising amino acid residues. Primary destabilising residues are directly recognised by the proteolytic machinery, whereas higher-order destabilising residues require prior modification before recognition. Although the eukaryotic N-end rule distinguishes between primary, secondary and tertiary destabilising residues, the prokaryotic version involves only primary and secondary destabilising residues, which are mostly different from the eukaryotic ones (Varshavsky, 1996; Mogk *et al.*, 2007). Also, the enzyme activities mediating the conversion of higher-order destabilising residues into primary destabilising residues differ between prokaryotes and eukaryotes. In bacteria, the enzyme leucyl/phenylalanyl-tRNA-protein transferase conjugates Leu or Phe to the secondary destabilising amino acid residues Arg or Lys using tRNA-Leu or tRNA-Phe as substrate (Shrader *et al.*, 1993). In eukaryotes, tertiary destabilising residues can be converted to secondary destabilising residues through the action of N-terminal aminohydrolase (acting on N-terminal Asn or Gln residues) or chemical modification of cysteine. Secondary destabilising residues can be converted to primary destabilising residues via N-terminal arginylation mediated by an arginyl-tRNA-protein arginyltransferase.

Whether or not an N-end rule exists in the chloroplast is currently unknown. Transgenic experiments have revealed that accumulation of recombinant proteins in plastids is controlled to a large extent at the level of protein stability (e.g. Birch-Machin *et al.*, 2004; Bellucci *et al.*, 2005; Zhou *et al.*, 2008; Oey *et al.*, 2009a,b;). In a few reported cases, N-terminal fusions resulted in serendipitous stabilisation of otherwise unstable recombinant proteins (Ye *et al.*, 2001; Herz *et al.*, 2005; Lenzi *et al.*, 2008), but the molecular basis for this remains unknown. In order to unravel the determinants of plastid protein (in)stability, we sought to develop an experimental system suitable for analysing the effects of N- and C-terminal modifications on the stability of a model protein. In the absence of faithful *in vitro* systems or workable transient transformation-based assays to study plastid protein degradation, stable transformation of the plastid genome is the only method that can currently be used to reliably measure protein stability in chloroplasts. Unfortunately, the generation of plants with transgenic chloroplasts (referred to as transplastomic plants) is technically demanding and involves time-consuming and laborious techniques (Maliga, 2004), thereby severely limiting the number of constructs that can be introduced into plants. Combination of a number of small improvements in vector construction, transformation procedures and selection and regeneration protocols (Bock, 2004; Wurbs *et al.*, 2007) has recently made it possible to produce transplastomic plants on a somewhat larger scale.

Here, we have used plastid transformation in the model plant tobacco to analyse the impact of N- and C-terminal sequences on chloroplast protein stability. We report that major determinants of the protein half life are located in the N-terminus, and the C-terminus is relatively unimportant for plastid protein stability. Moreover, systematic testing of all 20 proteinogenic amino acids in the position following the initiator Met provides evidence for an important role of the penultimate N-terminal amino acid residue in determining protein half lives.

## Results

### Design of *in vivo* reporters of protein stability in plastids

**Figure 1:**
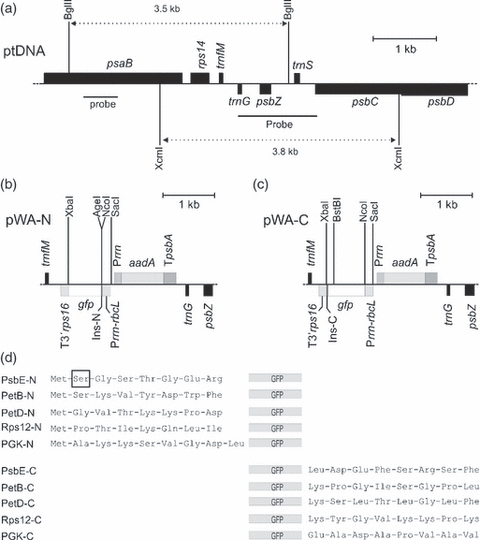
Construction of transformation vectors to analyse determinants of protein stability in plastids.(a) Physical map of the targeting region in the plastid genome from which the chloroplast transformation vectors were derived. The transgenes are targeted to the intergenic spacer between the *trnfM* and *trnfG* genes. Restriction sites used for RFLP analysis and their corresponding fragment sizes in the wild-type are indicated. Hybridisation probes are indicated by horizontal bars. Genes above the line are transcribed from the left to the right, those below the line are transcribed in the opposite direction.(b, c) Maps of the vector used to test N-terminal (b) and C-terminal (c) determinants of protein stability. Ins-N represents the sequence for the eight or nine N-terminal amino acids of five chloroplast proteins. Ins-C represents the sequence for the eight C-terminal amino acids of five chloroplast proteins. Relevant restriction sites used for cloning and/or exchange of N- or C-terminal sequence motifs are indicated. Vectors pWA-N and pWA-C harbour the selectable marker gene *aadA* (Svab and Maliga, 1993) driven by a chimeric rRNA operon promoter (P*rrn*) and the 3′ UTR from the *psbA* gene (T*psbA*). The *gfp* reporter gene is controlled by the ribosomal RNA operon promoter (P*rrn*), a 5′ UTR containing an *rbcL*-derived Shine–Dalgarno sequence, and the 3′ UTR from the *rps16* gene (T3′*rps16*).(d) Schematic representation of the N- and C-terminal fusion constructs, with the amino acid sequences fused to GFP given using the three-letter code. The codon in the PsbE N-terminus that was systematically modified is boxed. For technical reasons (codon constraints), the PGK N-terminus comprises nine amino acids.

Key determinants of protein stability in both eukaryotes and prokaryotes are located at the N-terminus. To test whether or not N-terminal sequences are also major determinants of protein stability in plastids, a reporter gene cassette was constructed that allowed easy manipulation of the N-terminus of the green fluorescent protein, GFP (Figure 1a,b). As we wished to investigate the N-terminus of a native plastid protein, we chose the plastid genome-encoded PsbE protein and fused its N-terminus in-frame to GFP (Figure 1b). PsbE is the α-subunit of cytochrome b_559_, an essential structural and functional component of photosystem II (Pakrasi *et al.*, 1991). Its eight amino acid N-terminal domain was chosen for two reasons. First, the N-terminal domain is soluble and exposed to the stromal side of the thylakoids (Pakrasi *et al.*, 1991). Second, the N-terminus of the PsbE protein had been determined by protein sequencing and was found to be an unmodified Ser generated by N-terminal Met excision (Sharma *et al.*, 1997). Moreover, the soluble N-terminal region including the Ser is highly conserved among plants (Kudla and Bock, 1999). Plasmid pWA-N (Figure 1b) was used to systematically exchange the Ser codon with the codons for all proteinogenic amino acids, resulting in 20 transformation vectors that were identical except for the codon downstream of the initiator Met of the *psbE–*GFP fusion gene. The resulting plasmids were named pWA-N-Ala, pWA-N-Arg, etc., and the transgenic organisms generated using them were simply named using the three-letter code for the relevant amino acid.

Whether the major (in)stability determinants of plastid proteins reside in the N-terminus, the C-terminus or both is currently unknown. In bacteria, it is known that C-terminal sequence motifs can also trigger protein degradation (Dulebohn *et al.*, 2007). Therefore, we also wished to determine the importance of N-terminal versus C-terminal sequences for chloroplast protein stability. To this end, a second transformation vector (pWA-C; Figure 1c) was constructed that facilitated manipulation of the C-terminus of the GFP reporter. We chose five chloroplast proteins to test the impact of their N-terminal and C-terminal sequences on the stability of the reporter protein GFP. The selection criteria for the proteins included (i) confirmed sequences of the N- and C-terminal regions (by direct protein sequencing), and (ii) solubility and stromal exposure of the N- and C-terminal domains. In addition to the PsbE protein used to determine the impact of the penultimate N-terminal amino acid on protein stability, the following proteins were selected: PetB (the cytochrome *b* subunit of the cytochrome *b*_6_*f* complex), PetD (subunit IV of the cytochrome *b*_6_*f* complex), Rps12 (ribosomal protein S12 of the 30S subunit of the plastid ribosome) and PGK (phosphoglycerate kinase, a Calvin cycle enzyme). PsbE, PetB, PetD and Rps12 are encoded by the plastid genome, but PGK is encoded by the nuclear genome and its N-terminus is generated by post-import cleavage of the transit peptide that directs the protein to the chloroplast. We used the sequences of the N-terminal eight amino acids from PsbE, PetB, PetD and Rps12 to generate N-terminal fusions with GFP in vector pWA-N. Due to codon constraints caused by requirement for an *Nco*I-compatible overhang for cloning, nine amino acids of the mature N-terminus were used for PGK. Likewise, the sequences of the C-terminal eight amino acids were used to produce C-terminal fusions with GFP in vector pWA-C (Figure 1d). The resulting plasmids were named pWA-N-PsbE, pWA-N-PetB, pWA-N-PetD, pWA-N-Rps12, pWA-N-PGK, pWA-C-PsbE, pWA-C-PetB, pWA-C-PetD, pWA-C-Rps12 and pWA-C-PGK. For brevity, the resulting transgenic organisms were simply named PsbE-N, PetB-N, PetD-N, Rps12-N, PGK-N, PsbE-C, PetB-C, PetD-C, Rps12-C and PGK-C.

All 30 cassettes were cloned into a plastid transformation vector suitable for targeting the transgenes to a defined location in the plastid genome by homologous recombination (Ruf *et al.*, 2001). The vector contains a chimeric spectinomycin resistance gene that facilitates the selection of plants with transgenic chloroplast genomes (transplastomic plants; Svab and Maliga, 1993). As plastids are derived from cyanobacteria and have retained a prokaryotic-type gene expression machinery, plastid expression elements (promoters and untranslated regions, UTRs) are usually active in eubacteria. This allowed us to test identical reporter gene constructs in *Escherichia coli*, and hence perform a side-by-side comparison of protein stability determinants between bacteria and plastids.

### Analysis of protein stability in *E. coli*

**Figure 2:**
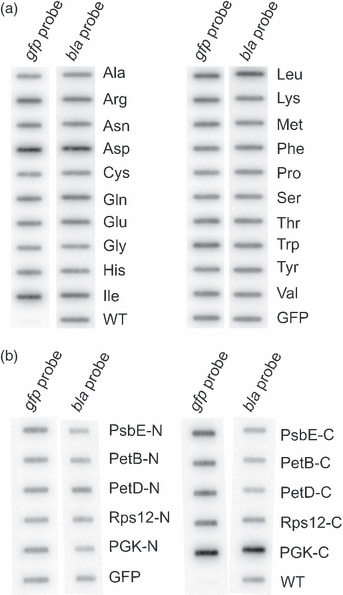
Analysis of RNA accumulation in *E. coli* by slot-blot assays. (a) Constructs harbouring mutations of the penultimate N-terminal amino acid residue.(b) Constructs for testing N- and C-terminal determinants of protein stability. Samples of 100 ng total RNA were blotted and hybridised to a *gfp*-specific probe, and, as a control for equal loading, to a *bla*-specific probe detecting transcripts from the ampicillin resistance gene localised on the same plasmid vector.WT, negative control (a bacterial strain transformed with a control vector lacking a *gfp* gene); GFP, positive control (a bacterial strain expressing unfused GFP).

To analyse the effects of the 20 mutations of the penultimate N-terminal amino acid residue as well as the N- and C-terminal fusions of GFP to the various plastid proteins in bacteria, all 30 constructs were introduced into *E. coli* cells. As any sequence modification of the *gfp* transcript can potentially affect mRNA stability, we wished to exclude the possibility that differences in *gfp* mRNA accumulation interfere with the assessment of protein stability as determined by GFP accumulation. We therefore compared *gfp* mRNA accumulation for all constructs (Figure 2). To obtain reliable quantitative data, the *gfp* hybridisation signal was normalised to the *bla* mRNA hybridisation signal. The *bla* gene, which encodes the ampicillin resistance-conferring enzyme β-lactamase, is also located on the transformation vector and is therefore a suitable control (i.e. copy number-independent) for mRNA stability. Quantification of hybridisation signal intensities and normalisation of the *gfp* signal to the *bla* signal revealed similar *gfp* mRNA accumulation levels for all constructs (Figure 2). This shows that none of the sequence modifications in our 30 constructs affect *gfp* transcript stability, and suggests that accumulation of GFP can serve as a suitable indicator of protein stability. Furthermore, all bacterial strains were fluorescent, indicating proper protein folding and confirming that GFP tolerates the N- and C-terminal fusions.

**Figure 3:**
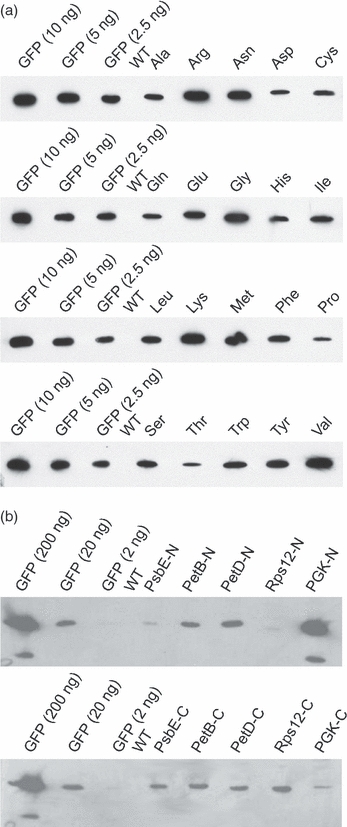
Western blot analysis to determine accumulation of the GFP reporter in *E. coli* using an anti-GFP antibody.(a) Constructs harbouring mutations of the penultimate N-terminal amino acid residue. For each transformed strain, 6 μg total soluble protein was loaded.(b) Constructs for testing N- and C-terminal determinants of protein stability. For each construct, 8 μg total soluble protein was loaded.WT, protein extract from bacteria transformed with a control vector lacking a *gfp* gene. For quantitative assessment of protein accumulation, a dilution series of purified recombinant GFP was included. For clarity, all GFP-containing samples are separated from each other by an empty lane. Data were confirmed by analysis of three biological replicates. Equal loading was further confirmed by Coomassie staining of the high-molecular-weight region of the gel (which was not blotted). To control for technical variation, the experiments were repeated three times and similar results were obtained.

We next investigated GFP accumulation in all *E. coli* strains using a specific antibody. As expected (Tobias *et al.*, 1991), analysis of the 20 constructs harbouring mutations of the penultimate N-terminal amino acid residue revealed substantial differences in protein accumulation levels (Figure 3a). For example, Thr and Pro conferred relatively low protein stability, whereas Val, Lys and Gly and Arg gave rise to very stable proteins. Interestingly, comparison of the five N-terminal fusion constructs showed even stronger differences in protein accumulation. While the N-terminal sequence of the PGK protein triggered very high levels of GFP accumulation, the Rps12–GFP and PsbE–GFP fusion proteins were barely detectable (Figure 3b). In contrast, the effect of the C-terminal sequences was much less pronounced. Four of the five plastid protein sequences conferred nearly identical GFP accumulation levels, and only the PGK fusion (strain PGK-C) resulted in approximately threefold lower protein stability than the other fusions (Figure 3b).

### Generation of transplastomic plants expressing protein stability reporters

In order to analyse plastid protein stability *in planta*, all 30 constructs were introduced into plants by stable transformation of the chloroplast genome (Svab and Maliga, 1993; Bock, 2001). Chloroplast transformation was performed by particle bombardment followed by selection of spectinomycin-resistant cell lines. Spectinomycin resistance is conferred by the chimeric *aadA* gene (Svab and Maliga, 1993) driven by plastid expression signals (Figure 1). For each construct, several putative transplastomic lines were obtained and subjected to additional rounds of regeneration under antibiotic selection to eliminate residual wild-type copies of the plastid genome and isolate homoplasmic lines (Bock, 2001).

**Figure 4:**
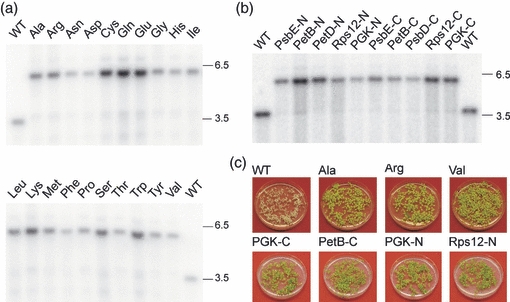
Molecular and genetic analysis of transplastomic tobacco lines.(a) Southern blot analysis of transplastomic lines generated with constructs harbouring mutations of the penultimate N-terminal amino acid residue. Total cellular DNA was digested with *Bgl*II and hybridised to a radiolabelled probe detecting the region of the plastid genome that flanks the transgene insertion site (see Figure 1a). WT, wild-type control. Fragment sizes of the molecular weight marker are indicated at the right in kb.(b) Southern blot analysis of transplastomic lines produced to test N- and C-terminal determinants of protein stability. DNA samples were digested with *Xcm*I. The sizes of hybridising bands are indicated on the right (in kb). The size difference of 2.3 kb between the hybridisation signals in the wild-type and the transplastomic lines corresponds to the combined size of the two transgene cassettes (Figure 1a). WT, wild-type control. Fragment sizes of the molecular weight marker are indicated at the right in kb.(c) Representative examples of seed tests to confirm homoplasmy of transplastomic lines. Seeds from the wild-type (WT) and seven selfed transplastomic plants were germinated on medium with spectinomycin. The lack of segregation of the antibiotic resistance in the T_1_ generation confirms the homoplasmic state of all transplastomic lines.

Successful plastid transformation and integration of the transgenes into the chloroplast genome by homologous recombination was verified by DNA gel-blot analyses (Figure 4a,b). As observed in previous plastid transformation experiments targeting the same genomic region (e.g. Wurbs *et al.*, 2007; Oey *et al.*, 2009b), the transplastomic lines also showed, in addition to the hybridisation signal for the transgenic plastid genome, a faint signal of wild-type size (Figure 4a,b). Previous work established that these weak wild type-like hybridisation signals do not represent heteroplasmy of the plastid genome, but instead are derived from so-called promiscuous DNA of plastid origin that is present in the tobacco nuclear genome (Hager *et al.*, 1999; Ruf *et al.*, 2000). To confirm homoplasmy of our transplastomic lines, all plants were grown to maturity, and seeds were obtained and analysed by inheritance tests. As plastid genes are maternally inherited and only few plastids are transmitted through seeds (Ruf *et al.*, 2007), large-scale phenotypic analysis of T_1_ seedlings raised on antibiotic-containing medium provides a highly sensitive assay of homoplasmy (Svab and Maliga, 1993; Bock, 2001). In these tests, all lines displayed a homogeneous population of antibiotic-resistant T_1_ seedlings, confirming that they are homoplasmic (Figure 4c).

### RNA accumulation and translation in transplastomic plants

**Figure 5:**
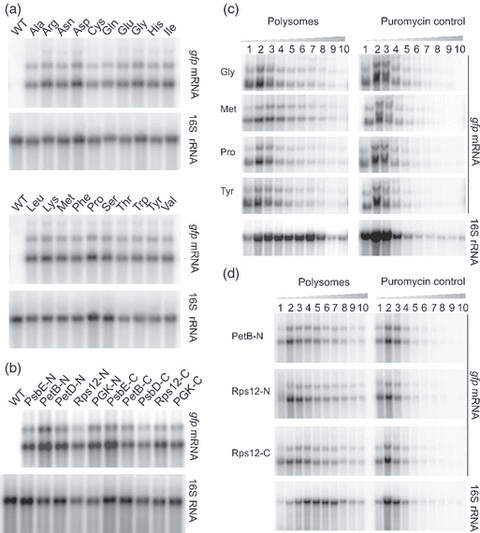
Analysis of transcript accumulation and ribosome association of mRNAs from *gfp* fusion genes in plastids.(a) RNA accumulation in transplastomic plants transformed with constructs harbouring mutations of the penultimate N-terminal amino acid residue.(b) RNA accumulation in plants transformed with constructs for testing N- and C-terminal determinants of protein stability. To control for equal loading, blots were first hybridised to a *gfp* probe, then stripped and re- hybridised to a 16S rRNA probe. The *gfp* probe detects two major transcript species. The lower band (0.9 kb) represents mature monocistronic *gfp* mRNA, and the upper band most likely represents a stable read-through transcript (Zhou *et al.*, 2007, 2008).(c) Analysis of polysome loading in selected transplastomic lines generated with constructs harbouring mutations of the penultimate N-terminal amino acid residue.(d) Analysis of polysome loading in selected transplastomic lines transformed with constructs for testing N- and C-terminal determinants of protein stability.Collected fractions from the sucrose density gradients were numbered from the top to the bottom. Ribosome distribution in the gradients is revealed by hybridisation to a 16S rRNA probe. Equal aliquots of extracted RNAs from all fractions were separated by denaturing agarose gel electrophoresis, blotted and hybridised to radiolabelled probes specific for *gfp* and 16S rRNA. The wedges above each blot indicate the gradient in sucrose density (from low to high). As a control, a sample was treated with puromycin to cause dissociation of ribosomes from the mRNAs. To control for technical variation, the experiments were repeated three times and identical results were obtained.

To rule out the possibility that some of the sequence manipulations at the 5′ or 3′ end of the *gfp* coding region affect transcript stability, accumulation of the *gfp* mRNA was analysed in all transplastomic lines. Quantification of the *gfp* hybridisation signal and normalisation to the plastid 16S rRNA signal revealed similar *gfp* mRNA accumulation levels in all 30 transplastomic lines (Figure 5a,b), indicating that none of the sequence modifications had resulted in altered *gfp* transcript levels. As it is also conceivable that sequence elements within the coding region could affect translational efficiency, we next analysed mRNA association with ribosomes. As pulse-chase experiments are difficult to perform with multicellular organisms, polysome loading analyses were performed to assess translation (Barkan, 1998; Kahlau and Bock, 2008). Polysomes (mRNAs loaded with ribosomes) can be separated from free mRNAs in sucrose density gradients, and the distribution of a given mRNA across the gradient fractions correlates with ribosome number, in that the more ribosomes are associated with an mRNA molecule the deeper it migrates into the gradient.

Based on preliminary data on protein accumulation, transplastomic lines spanning a wide range of GFP expression levels (see below) were selected for polysome analysis. When the distribution of the *gfp* mRNA in fractionated polysome gradients was compared, no differences in mRNA loading with ribosomes was detected between the transplastomic plant lines analysed (Figure 5c,d). This indicates that polysome loading is very similar and independent of the sequence manipulations in the coding region.

### Analysis of protein stability in transplastomic plants

**Figure 6:**
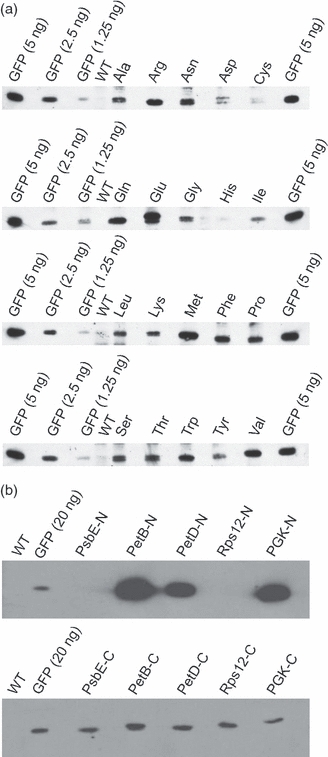
Western blot analysis to determine GFP accumulation in transplastomic tobacco plants using an anti-GFP antibody.(a) GFP accumulation in plants transformed with constructs harbouring mutations of the penultimate N-terminal amino acid residue. For each plant line, 20 μg total soluble protein was loaded. For quantitative assessment of protein accumulation, purified recombinant GFP was loaded at two dilutions.(b) GFP accumulation in plants transformed with constructs for testing N- and C-terminal determinants of protein stability. For each plant line, 5 μg total soluble protein was loaded. For clarity, all GFP-containing samples were separated from each other by an empty lane. The reproducibility of the data was confirmed by analysis of three biological replicates. Equal loading was further confirmed by Coomassie staining of the high-molecular-weight region of the gel (which was not blotted) and assessing the amount of large subunit of Rubisco (RbcL). WT, wild-type tobacco.

**Figure 7:**
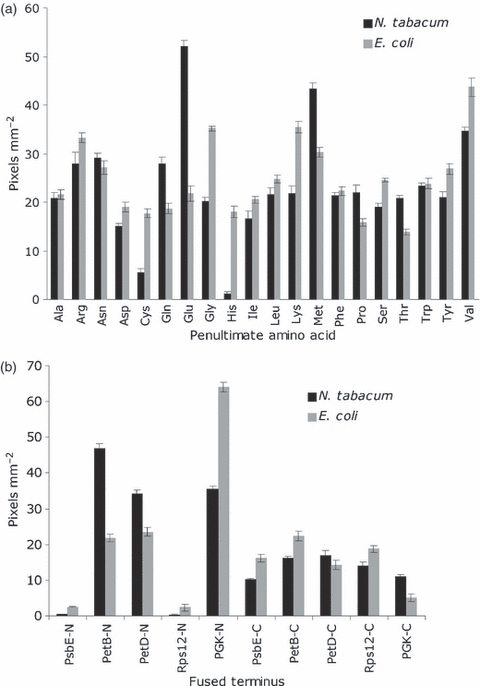
Quantification of GFP accumulation in *E. coli* and tobacco plastids.Three biological replicates of all blots were quantified using imagej software (http://rsb.info.nih.gov/ij/). The standard deviation is indicated by error bars.(a) Protein accumulation in plants transformed with constructs harbouring mutations of the penultimate N-terminal amino acid residue.(b) Protein accumulation in plants transformed with constructs for testing N- and C-terminal determinants of protein stability.

Our finding that *gfp* mRNA accumulation levels and polysome association are comparable in the transplastomic lines and not influenced by the sequence stretches fused to the 5′ or 3′ end of the *gfp* coding region made it clear that any difference observed in GFP accumulation is probably due to a difference in protein turnover. Consequently, GFP accumulation levels serve as a reporter of protein stability in plastids. Chloroplast-localised GFP fluorescence was detectable in the transplastomic lines, indicating proper protein folding of GFP *in planta*. GFP accumulation was not high enough to be directly detectable by Coomassie staining. We therefore measured protein accumulation in the 30 transplastomic lines using an anti-GFP antibody (Figure 6a,b). To exclude biological or technical variation, all results were confirmed using three biological replicates (Figure 7).

Interestingly, analysis of the mutations in the penultimate N-terminal amino acid residue revealed strong differences in GFP accumulation. The protein accumulated to the highest levels in the Glu, Met and Val lines (Figures 6a and 7a). Arg, Asn and Gln also triggered relatively high GFP accumulation levels. In contrast, GFP was barely detectable in the Cys and His plants, suggesting that these amino acids are strongly destabilising. Asp and Ile also appear to be relatively destabilising amino acids, but much less so than Cys and His (Figures 6a and 7a). The other amino acids conferred intermediate levels of GFP accumulation.

**Figure 8:**
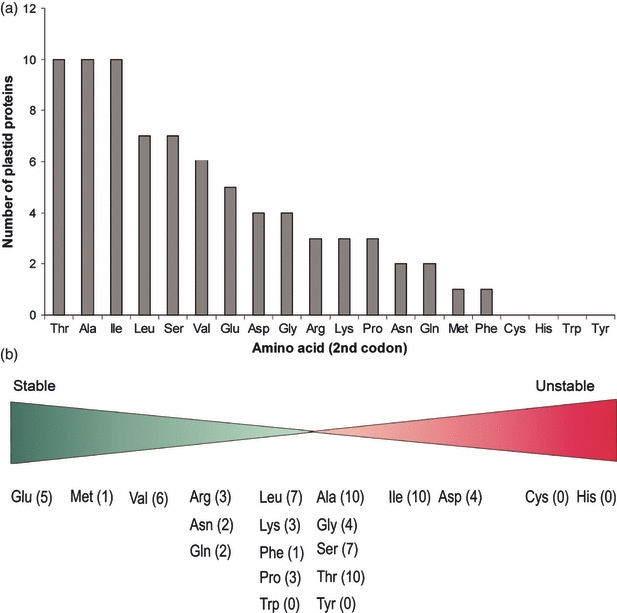
Protein stability with respect to the N-termini of plastid proteins.(a) Frequency of occurrence of each of the 20 proteinogenic amino acids after the initiator methionine in plastid-encoded proteins.(b) Relationship between protein stability and the frequency of occurrence of the 20 proteinogenic amino acids as penultimate N-terminal residues in plastid proteins.

Having established that the penultimate N-terminal amino acid residue has a strong impact on the half life of GFP expressed in the chloroplast, we wished to investigate whether or not additional determinants of protein stability reside in the N- or C-terminal sequences. To this end, we compared protein accumulation in the transplastomic lines expressing GFP N- or C-terminally fused to the termini of five plastid proteins. Interestingly, no strong differences in GFP accumulation levels were observed between the five C-terminal GFP fusions (Figure 6b), indicating that C-terminal amino acid sequences have little influence on protein half life. The PGK and PsbE C-termini conferred only moderately lower protein accumulation levels compared with the other three C-termini (Figure 7b). In contrast, the N-terminal fusions showed strong differences in protein stability. While the PetB-N, PetD-N and PGK-N lines accumulated very high levels of GFP, protein accumulation was much lower in the PsbE-N and Rps12-N lines (Figures 6b and 7b). It is important to note that these strong differences in protein accumulation conferred by the various N-terminal sequences cannot be explained by the identity of the penultimate amino acid alone. The penultimate N-terminal amino acid residues are Ser in PsbE-N, Ser in PetB-N, Gly in PetD-N, Pro in Rps12-N and Ala in PGK-N (Figure 1d). All these amino acids (Ser, Gly, Ala and Pro) conferred comparable levels of protein accumulation in the single amino acid mutation constructs (Figures 6a, 7 and 8b). Moreover, despite the presence of identical penultimate N-terminal amino acids (Ser) in PetB-N and PsbE-N, the two proteins differed considerably in terms of their stability (Figure 6b). This suggests that, in addition to the identity of the penultimate N-terminal amino acid residue, additional major determinants of plastid protein (in)stability reside in the most N-terminal part of the protein. The plastid PsbE and Rps12 proteins are not known to be particularly unstable *in vivo*, and it is possible that their N-termini are protected by neighbouring proteins in the multi-protein complexes in which they reside (Rps12 in the small subunit of the ribosome and PsbE in photosystem II; Barber *et al.*, 1997).

It is well established that the efficiency of post-translational removal of the initiator Met in both prokaryotes and eukaryotes depends on the identity of the penultimate amino acid residue. For example, it is known that bulky amino acid residues tend to impair cleavage of the initiator Met (Giglione *et al.*, 2004; Millán *et al.*, 2007). N-terminal sequencing of 59 plastid-encoded proteins revealed that 33 of them undergo efficient N-terminal Met excision (Giglione *et al.*, 2004; http://www.isv.cnrs-gif.fr/tm/maturation/images/chloro.html) but the others remain unprocessed (retaining either Met or N-formyl-Met as the N-terminal amino acid) or undergo more extensive N-terminal processing. Although N-terminal Met excision had been confirmed (for the native chloroplast proteins) by protein sequencing (http://www.uniprot.org/) for all plastid N-termini used in this study, it is likely that some of the amino acid exchanges in the 20 constructs with mutations of the penultimate N-terminal amino acid residue result in inefficient N-terminal Met removal. This could be the case in constructs where the initiator Met is followed by a bulky amino acid that is known to impair Met removal (and sometimes also deformylation) (Giglione *et al.*, 2004). One such example is glutamate, which greatly inhibits N-terminal processing in all three plastid genome-encoded proteins so far characterised in which Glu succeeds the initiator Met: PsaB (which retains Met), PsbM and PsbT (both of which retain fMet) (Giglione *et al.*, 2004). Therefore, the GFP fusion protein was purified from a Glu transplastomic line (by GFP affinity chromatography using an anti-GFP antibody coupled to magnetic beads) and subjected to mass spectrometry-based sequence determination. This analysis revealed that both the unprocessed form containing Met at the N-terminus and the processed form with Glu at the N-terminus were present (Figure S1), confirming inefficient Met excision if Glu follows the initiator Met. Attempts to investigate Met removal for other transplastomic lines have been unsuccessful, presumably due to the lower GFP accumulation levels and the difficult detection of the N-terminal peptide by mass spectrometry.

### The N-termini of plastid proteins

Having established that the penultimate N-terminal amino acid residue represents an important determinant of chloroplast protein (in)stability, we wished to identify putative substrates of the underlying protein degradation pathway in plastids. When analysing the penultimate N-terminal amino acids of all plastid genome-encoded proteins in tobacco, we found that the two strongest instability-conferring amino acids, Cys and His (Figures 6 and 7a), were not present in the penultimate position of any plastid-encoded protein (Figure 8). The most frequently occurring penultimate N-terminal amino acids were Thr, Ala and Ile (Figure 8a), which confer intermediate protein stability (with Ile causing slightly lower stability than Thr and Ala; Figures 6, 7a and 8b). A substantial number of proteins also carried the three most strongly stabilising amino acids Glu, Met and Val (Figure 8). It is noteworthy that both Glu and Val have been reported to inhibit N-terminal Met excision (Giglione *et al.*, 2004), as confirmed for Glu by our mass spectrometric sequence analysis. This raises the possibility that the high protein stability in the Glu and Val transplastomic lines is at least partly due to inefficient Met removal. This conclusion is supported by the fact that 25 plastid-encoded proteins (out of 59 analysed) were found to retain their initiator Met (http://www.isv.cnrs-gif.fr/tm/maturation/images/chloro.html).

In the absence of plastid-encoded proteins with destabilising Cys or His residues, we next considered the possibility that Cys and His serve as protein degradation signals for nuclear-encoded proteins targeted to plastids. The N-termini of imported proteins are generated by post-import proteolytic processing, which removes the N-terminal transit peptide from the protein. Previous work has established that the cleavage site is defined by the loosely conserved sequence motif (Val/Ile)-X-(Ala/Cys)↓Ala (Gavela and von Heijne, 1990). Consequently, Ala is the by far most frequently occurring amino acid at the N-terminus of nuclear-encoded plastid proteins, a finding that was confirmed in a recent comprehensive analysis of the chloroplast proteome (Zybailov *et al.*, 2008). N-terminal amino acids occurring at lower frequencies in a published set of proteins with experimentally determined transit peptide cleavage sites included Ser, Thr, Met and Lys (Gavela and von Heijne, 1990). None of the proteins in the set has His at the N-terminus and only a single protein has Cys. Interestingly, this single protein with Cys at the N-terminus is nitrite reductase (Gavela and von Heijne, 1990), a highly regulated chloroplast enzyme whose levels decline strongly if the enzyme is not needed (e.g. in the presence of sufficient ammonium; (Crete *et al.*, 1997). This suggests that nitrite reductase is a possible physiological substrate of an N-terminus-dependent protein degradation pathway in plastids. Other potential substrates may be generated by oxidative damage-induced protein cleavage at His and Cys residues, as discussed below.

## Discussion

In this study, we have identified key determinants of protein stability in chloroplasts. Our data obtained with fusions of N- and C-terminal sequences from five plastid proteins to GFP provide strong evidence that the N-terminus harbours important information affecting the half life of the protein. Although the identity of the penultimate N-terminal amino acid residue is part of this stability information, additional, and perhaps more significant, stability determinants reside downstream, as revealed by the strong differences in stability conferred by N-termini with identical or similar penultimate N-terminal residues (Figures 1d and 6). We therefore propose that the stability of plastid proteins is determined by at least three factors: (i) the action of methionine aminopeptidase (the enzyme that removes the initiator Met and exposes the penultimate N-terminal amino acid residue with an efficiency that varies with sequence context), (ii) an N-end rule-like protein degradation pathway in which protein stability is dependent on the identity of the penultimate amino acid residue, and (iii) additional sequence determinants in the N-terminal region. However, based on the limited set of N- and C-termini tested in this study, we cannot exclude the possibility that C-terminal sequences play a more pronounced role in determining protein stability in certain plastid proteins than suggested by our data.

Previous work on N-end rule degradation in *E. coli* has mainly involved cleavage of reporter proteins, in which the N-terminus of the test protein was generated by post-translational proteolytic processing (e.g. by co-expressing ubiquitin–βGal fusions and the yeast de-ubiquitination enzyme Ubp1; Tobias *et al.*, 1991). Although this has the advantage of generating defined N-termini and excluding the influence of inefficient N-terminal Met excision in certain sequence contexts (Giglione *et al.*, 2004), it has the disadvantage of producing artificial N-termini, whose stability properties may not be relevant for the *in vivo* half lives of endogenous proteins. For example, Leu, a highly destabilising amino acid according to the prokaryotic N-end rule (Tobias *et al.*, 1991), often protects proteins from N-terminal Met excision (Giglione *et al.*, 2004). Therefore, the presence of an N-terminal Leu may have little significance in determining the turnover time of full-length (unprocessed) bacterial proteins, and may be more relevant to determining the half lives of protein fragments generated by proteolytic cleavage.

The physiological function of the prokaryotic N-end rule is unclear. *E. coli* strains lacking the ClpS adapter protein have no detectable mutant phenotype and only very few *in vivo* substrates of the bacterial N-end rule pathway have been identified to date (Mogk *et al.*, 2007; Ninnis *et al.*, 2009). The aim of this work was to investigate the stability properties of native plastid N-termini, and therefore, instead of using cleavable fusion proteins, the 20 protein versions were produced by modifying the first codon after the initiator Met. When the constructs were analysed in *E. coli*, some deviations from the N-end rule established using cleavable fusion proteins (Tobias *et al.*, 1991) were observed. For example, Leu, Phe and Lys result in relatively stable proteins (Figure 3), most probably due to protection of the N-terminus by inefficient removal of the initiator Met (Giglione *et al.*, 2004). It is also possible that the dependence of bacterial N-end rule degradation on the sequence context and/or conformation of the protein accounts for some of the observed differences. Consistent with the published bacterial N-end rule (Tobias *et al.*, 1991), Val, Gly and Met in the penultimate position yielded stable proteins in our tests in *E. coli* (Figures 3 and 7). The lowest stability in our analyses was observed with Thr and Pro. While Thr at the N-terminus was previously reported to confer stability, Pro could not be tested due to the low rate of Ubp1-mediated de-ubiquitination of the ubiquitin–βGal fusion protein with Pro at the cleavage site (Tobias *et al.*, 1991).

When comparing protein stabilities in *E. coli* and plastids, significant differences were apparent (Figures 3, 6 and 7). For example, two of the most strongly destabilising amino acids in *E. coli*, Pro and Thr, confer intermediate protein stability in plastids. Conversely, Gly, a stabilising amino acid in *E. coli*, confers comparably low protein stability in plastids. However, there are also similarities between the two systems. Proteins with a retained N-terminal Met, which are known to be very stable in bacteria (Román-Hernández *et al.*, 2009), also appear to be very stable in plastids (Figures 3, 6 and 7). Likewise, Val results in stable proteins in both systems. However, as the codon most frequently used by the chloroplast translation machinery was used for all 20 amino acids, and the codon usage in plastids and *E. coli* is different, we cannot entirely exclude the possibility that GFP accumulation from some constructs in *E. coli* is influenced by use of unfavourable codons. Assessment of the triplets used in our constructs in relation to the codon usage in *E. coli* (http://www.kazusa.or.jp/codon/) revealed that we used only one codon that is rare in *E. coli* (i.e. used at a frequency of less than 10%): AGA for arginine (used for only 2% of the Arg codons in *E. coli* K12). However, as Arg resulted in high GFP accumulation in *E. coli* (Figure 3a), we believe that codon usage does not appreciably influence the results of our study.

Consistent with the idea that the N-terminus-dependent protein degradation pathway in plastids is not fully homologous to the bacterial one, we have been unable to identify a candidate gene for a key bacterial N-end rule enzyme, leucyl/phenylalanyl-tRNA-protein transferase (L/F transferase), in the fully sequenced genome of the model plant *Arabidopsis thaliana*. Similar searches for homologues of Bpt, another prokaryotic aminoacyl transferase that was recently discovered in the human pathogen *Vibrio vulnificus* (Graciet *et al.*, 2006), revealed only two Arabidopsis genes with limited similarity, both of which are annotated as arginyl-tRNA-protein arginyltransferases of the eukaryotic N-end rule pathway. For one of the two genes, this function has also been confirmed experimentally (Yoshida *et al.*, 2002).

In addition to the L/F transferase, the Clp protease components ClpA/P and ClpS play a crucial role in the bacterial N-end rule pathway (Tobias *et al.*, 1991; Erbse *et al.*, 2006; Wang *et al.*, 2008; Schmidt *et al.*, 2009). It seems reasonable to speculate that the chloroplast homologue of the bacterial Clp protease could be also involved in degrading plastid proteins with unstable N-terminal sequences. However, although plastids possess numerous isoforms of subunits of the Clp protease core complex, the ClpS adapter protein that is considered to be an essential component of the N-end rule pathway in *E. coli* (Erbse *et al.*, 2006; Schmidt *et al.*, 2009) has not yet been identified in proteomics studies of chloroplast Clp protein complexes (Peltier *et al.*, 2001, 2004; Adam *et al.*, 2006). The Arabidopsis nuclear genome encodes a putative homologue of ClpS, which has been termed ClpT, but as this protein has not been found in plastid Clp complexes (Peltier *et al.*, 2004), its involvement in plastid protein degradation remains uncertain. Generation of knockdown lines for ClpT and other components of the plastid proteolytic machinery and subsequent crosses to our transplastomic lines expressing GFP versions with destabilising N-terminal sequences would be a promising experimental approach to test candidate components of a plastid N-terminus-dependent protein degradation pathway.

Why do Cys and His serve as protein degradation signals in plastids although they do not occur as penultimate N-terminal residues in plastid-encoded proteins (Figure 8)? A search for possible substrate proteins that are encoded in the nuclear genome and imported into plastids revealed that, among a set of imported proteins with experimentally determined N-termini (Gavela and von Heijne, 1990), only nitrite reductase carried a strongly destabilising amino acid residue (Cys) at its N-terminus. It is possible that, in other plastid proteins, destabilising N-terminal amino acids are generated through internal cleavage by specific proteases. Cys and His are special in that they represent very reactive amino acids and also frequently serve as ligands for the binding of co-factors, including the redox-active co-factors in the photosynthetic complexes. For example, both His and Cys are common haem ligands, and Cys is the major amino acid involved in coordinating iron–sulphur clusters. Their high reactivity may make internal Cys and His residues in plastid proteins frequent sites of photo-oxidative damage-induced cleavage. In fact, singlet oxygen, a reactive oxygen species generated as a by-product of photosynthesis (Krieger-Liszkay and Trebst, 2006), reacts most strongly with His residues (Okada *et al.*, 1996). Likewise, H_2_O_2_, the second major reactive oxygen species generated in photosynthesis, reacts with non-haem iron centres, making the Cys residues coordinating them probable targets of H_2_O_2_-induced protein cleavage (Miyao *et al.*, 1995; Okada *et al.*, 1996). It is well established that exposure of the D1 protein of photosystem II to singlet oxygen results in protein cleavage at highly specific sites, and His residues 215 and 272 have been suggested as the most probable targets (Okada *et al.*, 1996). We therefore hypothesise that Cys and His residues that are N-terminally exposed by oxidative damage-induced cleavage may be major physiological substrates of N-terminus-dependent protein degradation in plastids.

The uncovering of sequence determinants that influence the stability of plastid-encoded proteins also has important practical implications. There are considerable advantages of using the plastid genome rather than the plant’s nuclear genome for transgene expression in biotechnology, including the plastid’s potential for high-level accumulation of foreign proteins and the maternal inheritance of the plastid genome in most crops, which greatly reduces the risk of uncontrolled pollen spread of transgenes (Ruf *et al.*, 2007; reviewed by Maliga, 2004; Bock, 2007). Applications of plastid genome engineering in both basic research and biotechnology depend critically on the predictability of transgene expression rates and the possibility of adjusting protein accumulation to the desired level. Recent work has demonstrated that protein accumulation in transplastomic plants is to a large extent controlled post-translationally, at the level of protein stability (e.g. Birch-Machin *et al.*, 2004; Bellucci *et al.*, 2005; Zhou *et al.*, 2008; Oey *et al.*, 2009a,b;). Unfortunately, until now, the rules that determine protein stability and instability in plastids have been unknown, making the accumulation level of a given foreign protein to be expressed from the plastid genome totally unpredictable, and leaving the experimenter with no other option but use of trial and error. The dependence of protein stability on the identity of the penultimate N-terminal amino acid residue and identification of the PetB N-terminus as a sequence conferring exceptionally high protein stability (Figure 6a,b) provide guidelines and tools for designing plastid expression constructs to optimise foreign protein accumulation and stabilise recombinant proteins expressed from the plastid genome.

## Experimental procedures

### Plant material and growth conditions

Sterile tobacco (*Nicotiana tabacum* cv. Petit Havana) plants were obtained from surface-sterilised seeds germinated on agar-solidified MS medium (Murashige and Skoog, 1962) with 20 g/L sucrose. Homoplasmic transplastomic lines were rooted and propagated on the same medium. Rooted homoplasmic plants were transferred to soil and grown to maturity under standard greenhouse conditions with supplementary lighting of approximately 200 μE m^−2^ sec^−1^. T_1_ seedling phenotypes were analysed by germinating transplastomic seeds on MS medium containing spectinomycin (500 mg/L).

### Bacterial strains and growth conditions

*Escherichia coli* cells (One Shot® Top10F’; Invitrogen, http://www.invitrogen.com/) harbouring the various plasmid constructs were grown in YT medium (8 g/L bactotryptone, 5 g/L yeast extract, 5 g/L NaCl) with ampicillin (100 μg/ml) at 37°C under continuous shaking (180 rpm).

### Construction of transformation vectors

All vectors are based on the previously described plastid transformation vector pRB95 (Ruf *et al.*, 2001) and its derivatives (Zhou *et al.*, 2008). An expression cassette comprising the P*rrn* promoter, the gene 10 leader from phage T7 (G10L), the *gfp* coding region and the *rps16* 3′ UTR was assembled from previously described elements (Ruf *et al.*, 2007; Oey *et al.*, 2009a,b;). The P*rrn–*5′ UTR fragment was excised by digestion with *Nco*I and *Sac*I and replaced by the ribosomal RNA operon promoter and a 5′ UTR containing an *rbcL*-derived Shine–Dalgarno sequence (Svab and Maliga, 1993), resulting in plasmid pWA1. To generate a basic vector for mutagenesis of the penultimate amino acid, the first eight codons of the *psbE* coding region were inserted into the *Nco*I-digested and dephosphorylated vector pWA1 as annealed and phosphorylated synthetic oligonucleotides 5′-CATGGGAGGATCTACCGGTGAACG-3′ and 5′-CATGCGTTCACCGGTAGATCCTCC-3′ (restriction sites underlined). The synthetic fragments have *Nco*I overhangs on both sides and contain an internal *Age*I restriction site (underlined). The basic vector pWA-Gly contains glycine as the first amino acid after the initiator Met. To change the second codon of the *psbE–gfp* fusion gene, pairs of annealed synthetic oligonucleotides were inserted into the *Nco*I/*Age*I-digested vector pWA-Gly. The oligonucleotide pairs had the sequences 5′-CATGNNNGGATCTA-3′ and 5′-CCGGTAGATCCNNN-3′, where NNN indicates the codon to be changed. Annealing of the oligonucleotides generated *Nco*I and *Age*I overhangs for cloning into pWA-Gly. For each amino acid, the most preferred codon was chosen according to the codon usage table for the tobacco plastid genome (http://www.kazusa.or.jp/codon/). To test larger N-terminal sequences for their impact on protein stability, the sequences for the first eight amino acids of the PetD, PetB and Rps12 proteins and the first nine amino acids of the PGK protein were inserted into the *Nco*I-digested pWA-1 vector as annealed synthetic oligonucleotide pairs with *Nco*I overhangs. The oligonucleotide pairs used were 5′-CATGGGAGTAACTAAAAAACCTGA-3′ and 5′-CATGTCAGGTTTTTTAGTTACTCC-3′ for PetD, 5′-CATGTCTAAAGTATATGATTGGTT-3′ and 5′-CATGAACCAATCATATACTTTAGA-3′ for PetB, 5′-CATGCCTACTATTAAACAATAAAT-3′ and 5′-CATGATTTATTGTTTAATAGTAGG-3′ for Rps12, and 5′-CATGGCTAAAAAATCTGTAGGAGATCT-3′ and 5′-CATGAGATCTCCTACAGATTTTTTAGC-3′ for PGK. C-terminal sequences from the same plastid proteins were integrated at the 3′ end of the *gfp* coding region as *Bst*bI/*Xba*I-digested PCR products generated using the forward primer 5′-GAGTACAACTATAACTCACAC-3′ and one of the following gene-specific oligonucleotide primers: 5′-AAAATCTAGA**TTAAAATGATCGTGAAAATTCATCTAA-**TTTGTATAGTTCATCCATGCC-3′ for PsbE, 5′-AAAATCTAGA**TTA-AAATAATCCTAAAGTTAAAGATTT**TTTGTATAGTTCATCCATGCC-3′ for PetD, 5′-AAAATCTAGA**TTATAAAGGTCCAGAAATTCCAGGTTT**TTTGTATAGTTCATCCATGCC-3′ for PetB, 5′-AAAATCTAGA**TTATTTAGGTTTTTTTACTCCATATTT**TTTGTATAGTTCATCCATGCC-3′ for Rps12, and 5′-AAAATCTAGA**TTATACAGCTACAGGAGCATCAGC-TTC**TTTGTATAGTTCATCCATGCC-3′ for PGK (C-terminal sequence in bold, *Xba*I restriction site underlined).

### Transformation of chloroplasts

Plastid transformation was performed using the biolistic protocol (Svab and Maliga, 1993). Briefly, young leaves from aseptically grown tobacco plants were bombarded with plasmid DNA-coated 0.6 μm gold particles using a PDS1000He gun (Bio-Rad, http://www.bio-rad.com/) with a Hepta adaptor. Spectinomycin-resistant lines were selected on a modified MS medium containing 500 mg/L spectinomycin. Plastid transformants were identified by double resistance tests on medium containing both spectinomycin and streptomycin (500 mg/L each) (Bock, 2001). For each construct, several independent transplastomic lines were subjected to two to three additional rounds of regeneration on spectinomycin-containing medium to obtain homoplasmic tissue, and at least three lines per construct were subsequently analysed molecularly. Homoplasmy was confirmed by RFLP analyses and inheritance assays in which seeds were germinated on MS medium containing spectinomycin (500 mg/L).

### Isolation of nucleic acids and gel-blot analyses

Total plant DNA was isolated from fresh leaf tissue by a cetyltrimethylammoniumbromide-based method (Doyle and Doyle, 1990). Total cellular RNA was extracted from fresh leaf using the peqGOLD TriFast reagent (Peqlab GmbH, http://www.peqlab.de). RNA from *E. coli* was isolated using a NucleoSpin® Extract II kit (Macherey-Nagel, http://www.macherey-nagel.com). RNA samples were purified by treatment with RNase-free DNase I (Roche, http://www.roche.com) and tested for the absence of residual DNA by PCR. For Southern blot analysis, DNA samples (5 μg total DNA) were digested using *Bgl*II or *Xcm*I, separated by gel electrophoresis in 1% agarose gels, and transferred onto Hybond nylon membranes (GE Healthcare, http://www.gehealthcare.com) by capillary blotting using standard protocols. RNA samples were electrophoresed in formaldehyde-containing 1% agarose gels and blotted onto Hybond nylon membranes or transferred directly onto membranes using a slot-blot apparatus (SCIE-PLAS Ltd, http://www.scie-plas.co.uk). For slot blotting, RNA samples were diluted in 200 μl in DNase I buffer and heat-denatured. Hybridisations were performed at 65°C (Church and Gilbert, 1984). A 550 bp PCR product generated by amplification of a portion of the *psaB* coding region (Wurbs *et al.*, 2007) and a 283 bp PCR product covering a portion of the *psbZ* coding region [obtained using primers psbZ-F (5′-GCTGATAGAGGGATCAAAT-3′) and psbZ-R (5′-GGGTCATTTTGGTTTTGGG-3′)] were used as RFLP probes to verify plastid transformation and assess homoplasmy. An *E. coli* 16S rRNA-specific probe and a *gfp*-specific probe were produced as described previously (Neupert *et al.*, 2008). A *Dra*I restriction fragment from a pBluescript vector was used as the *bla*-specific probe, and a plastid 16S rRNA probe was generated by PCR using primers P16Srrn-F (5′-CAAGCGGTGGAGCATGTGG-3′) and P16Srrn-R (5′-GGCGGTGTGTACAAGGCCC-3′). Probes were purified by agarose gel electrophoresis after extraction of the DNA fragments of interest from excised gel slices using a GFX™ PCR kit (GE Healthcare), and then radiolabelled by random priming using [α-^32^P]dCTP.

### Isolation of polysomes

Polysome isolation was performed as described previously (Kahlau and Bock, 2008). Each gradient was separated into ten fractions for RNA gel-blot analysis. Control gradients with puromycin were analysed to identify polysome-containing fractions that lacked free mRNA and ribosomes. After phenol:chloroform extraction, the RNA was precipitated with ethanol.

### Protein isolation and Western blot analyses

Extraction of total soluble protein from plant material and from *E. coli* cells (harvested at an OD_600_ of approximately 1.0) was performed as described previously (Neupert *et al.*, 2008; Oey *et al.*, 2009a,b;). For quantitative comparison of soluble and total bacterial proteins, *E. coli* lysates (bacterial pellets solubilised in SDS–PAGE loading buffer) were also analysed. Protein samples were separated by electrophoresis on 15% SDS-containing polyacrylamide gels and blotted onto polyvinylidene difluoride membranes (Hybond-P; GE Healthcare). Membranes were treated with blocking buffer (20 mm Tris/HCl, pH 7.6, 137 mm NaCl, 0.1% Tween-20, 1% BSA) for 1 h, and then incubated for 1 h with polyclonal GFP antibody (JL-8; Clontech, http://www.clontech.com/) diluted 1:10 000 in buffer (20 mm Tris/HCl, pH 7.6, 137 mm NaCl, 0.1% Tween-20). Detection was performed using the ECL Plus detection system (GE Healthcare) and an anti-mouse secondary antibody (Sigma-Aldrich, http://www.sigmaaldrich.com/). To exclude technical or biological variation, all experiments were repeated at least three times. Equal loading was confirmed by Coomassie staining of the high-molecular-weight region of the gel using standard protocols and quantitatively assessing the amount of large subunit of Rubisco (RbcL) in all plant samples. Image quantification was performed using the Java software package imagej (http://rsb.info.nih.gov/ij/). The recombinant GFP signals were used for ECL exposure adjustment and normalisation between replicates.

### GFP purification and mass spectrometric analysis

GFP fusion proteins were purified from leaf material using the μMACS GFP isolation kit (Miltenyi Biotech GmbH, http://www.miltenyibiotec.com) with affinity chromatography columns from the same supplier. Total soluble protein extract (18 ml, obtained from approximately 700 mg leaf material per ml extraction buffer) was used for the chromatography. The eluted GFP fusion protein was further purified by gel electrophoresis and prepared for mass spectrometry by tryptic digestion as described previously (Rogalski *et al.*, 2008). Peptide preparation and mass spectrometric analysis were performed as described previously (Kierszniowska *et al.*, 2009). Fragment MS/MS spectra were extracted from raw data as DTA files using DTA supercharge version 1.19 (http://msquant.sourceforge.net), and searched for the N-terminal peptides using the Mascot algorithm (http://www.matrixscience.com). The search parameters included: 50 ppm precursor mass accuracy, 0.8 Da fragment mass tolerance, carbamidomethyl cysteine as a fixed modification, and oxidation of methionine as a variable modification. One missed cleavage was allowed.

## SUPPORTING INFORMATION

Figure S1. Fragment spectra of N-terminal peptides of the GFP fusion protein purified from a Glu transplastomic line.

Please note: As a service to our authors and readers, this journal provides supporting information supplied by the authors. Such materials are peer-reviewed and may be re-organised for online delivery, but are not copy-edited or typeset. Technical support issues arising from supporting information (other than missing files) should be addressed to the authors.
